# Genomic and Phenotypic Insights into Carbapenemase-Mediated Resistance and Clonal Diversity of *Pseudomonas aeruginosa* Clinical Isolates from Southern Brazil

**DOI:** 10.1007/s00284-026-05023-9

**Published:** 2026-06-18

**Authors:** Bruna Mezzomo Bejes, Marcelo Ricardo Vicari, Viviane Nogaroto, Larissa Bail, Lavinia Nery Villa Stangler Arend, Keite da Silva Nogueira, Sônia Alvim Veiga Pileggi, Felipe Francisco Tuon, Carmen Antonia Sanches Ito, Luiz Ricardo Olchanheski, Marcos Pileggi

**Affiliations:** 1 Programa de Pós-Graduação em Ciências Farmacêuticas, Universidade Estadual de Ponta Grossa, Av. Carlos Cavalcanti, 4748, Ponta Grossa, , 84030-900 Paraná Brazil; 2 Departamento de Biologia Estrutural, Molecular e Genética, Universidade Estadual de Ponta Grossa, Av. Carlos Cavalcanti, 4748, Ponta Grossa, , 84030-900 Paraná Brazil; 3 Departamento de Análises Clínicas e Toxicológicas, Universidade Estadual de Ponta Grossa, Av. Carlos Cavalcanti, 4748, Ponta Grossa, , 84030-900 Paraná Brazil; 4 Laboratório Central do Estado do Paraná, Rua Sebastiana Santana Fraga, 1395, São José dos Pinhais, , 83060- 500 Paraná Brazil; 5 Departamento de Patologia Básica do Hospital de Clínicas, Universidade Federal do Paraná, Rua Gen. Carneiro, 181, Curitiba, 80060-900 Paraná Brazil; 6 Laboratório de Doenças Infecciosas Emergentes, Escola de Medicina, Pontifícia Universidade Católica do Paraná, Rua Imaculada Conceição, 1155, Curitiba, , 80215-901 PR Brazil; 7 Departamento de Ciências Básicas da Saúde, Universidade Estadual de Maringá, Av. Colombo, 5790 – Zona 7, Maringá, 87020-900 Paraná Brazil

## Abstract

*Pseudomonas aeruginosa* is a major opportunistic pathogen associated with high morbidity in hospitalized patients due to its intrinsic and acquired resistance mechanisms. Carbapenem resistance, often mediated by the production of carbapenemase, poses a critical therapeutic challenge worldwide. This study investigated the genomic organization, molecular diversity, and plasmid-mediated dissemination of carbapenemase genes in *P. aeruginosa* isolates from hospitals in Paraná and Santa Catarina, Brazil, and explored their correlation with phenotypic resistance profiles. Eight isolates (80%) were classified as extensively drug-resistant (XDR), showing broad resistance to β-lactams, carbapenems, and β-lactam/β-lactamase inhibitor combinations. Multi-Locus Sequence Typing revealed a heterogeneous clonal structure, with ST1560 being the predominant type (30%). Multiple β-lactamase genes were identified, including chromosomal *bla*PDC variants, *bla*OXA-50, and carbapenemase genes *bla*SPM-1, *bla*IMP-16, *bla*IMP-1, *bla*VIM-2, *bla*KPC-2, and *bla*NDM-1. Notably, 40% of isolates carried plasmid-borne carbapenemase genes, indicating a potential for horizontal gene transfer. Isolate 20,783 exhibited high resistance despite lacking additional carbapenemase genes, suggesting alternative mechanisms such as efflux or porin loss. The predominance of XDR *P. aeruginosa*,which harbors diverse carbapenemases, including plasmid-mediated determinants, underscores the complexity of antimicrobial resistance in Brazilian hospitals. The coexistence of multiple resistance mechanisms, coupled with clonal heterogeneity, highlights the urgent need for integrated genomic surveillance and targeted infection control strategies to mitigate the spread of multidrug-resistant *P. aeruginosa* in clinical settings.

**Supplementary Information:**

The online version contains supplementary material available at 10.1007/s00284-026-05023-9.

## Introduction

*Pseudomonas aeruginosa* is a non-fermenting, Gram-negative bacterium that can form biofilms and produce virulence factors, including exotoxin A, elastase, proteases, and pyocyanin, which contribute to tissue damage and immune evasion [1]. Clinically, it is associated with pneumonia, urinary tract infections, surgical site infections, endocarditis, and septicemia, particularly in immunocompromised patients or those with prolonged catheter use, leading to high morbidity and extended hospital stays [1, 2]. Its lineage exhibits intrinsic resistance through low outer membrane permeability, efflux pumps, porin loss, and antibiotic-inactivating enzymes, and can acquire additional resistance genes, including extended-spectrum β-lactamases and carbapenemases, further limiting treatment options [3]. Resistant strains to all first-line antipseudomonal agents are classified as *P. aeruginosa* with difficult-to-treat resistance (DTR-PA) [4], spanning β-lactams/β-lactamase inhibitor, extended-spectrum cephalosporins, carbapenems, and fluoroquinolones [5, 6].

Carbapenems are last-line agents for multidrug-resistant *P. aeruginosa*. Still, resistance has increased due to the production of carbapenemases, including class A (*bla*KPC), class D (*bla*OXA), and class B metallo-β-lactamases (*bla*VIM, *bla*IMP, *bla*NDM, *bla*SPM), which are often carried on mobile genetic elements such as plasmids, integrons, and transposons [7–10]. Detection of these genes usually requires advanced molecular techniques, such as multiplex PCR or whole-genome sequencing, which are not always readily available in routine clinical practice. This study aims to characterize the genetic diversity and antimicrobial resistance profiles, with a focus on the dissemination of carbapenemase-encoding genes and their association with mobile genetic elements in *P. aeruginosa* isolates from hospital environments in Brazil.

## Materials and Methods

### Collection of CRPA Isolates

Carbapenem-resistant *P. aeruginosa* isolates were collected between January 2014 and September 2018 from healthcare institutions in the states of Paraná and Santa Catarina, Brazil. Bacterial identification was performed by matrix-assisted laser desorption/ionization time-of-flight mass spectrometry (MALDI-TOF VITEK^®^ MS, bioMérieux), following the manufacturer’s instructions. A total of 300 bacterial isolates were successfully obtained, of which 10 were selected for antimicrobial susceptibility profiling and whole-genome sequencing based on their resistance profiles to the novel β-lactam/β-lactamase inhibitor combinations ceftazidime-avibactam, ceftolozane-tazobactam, and imipenem-relebactam, which comprised the routine antimicrobial panel implemented in the participating laboratories for surveillance of carbapenem-resistant *P. aeruginosa*. The selection aimed to capture a diversity of phenotypic responses, including both resistant and susceptible/intermediate isolates, to enable comparative genomic analysis. This was an exploratory study designed to identify genetic determinants associated with differential susceptibility to these novel agents, rather than a population-based epidemiological survey. Consequently, the selected sample is not intended to represent the full epidemiological diversity of *P. aeruginosa* in the region. This study was approved by the Research Ethics Committee of Pontifícia Universidade Católica do Paraná under number 3.179.848. Authorization for access to genetic resources, in compliance with Brazilian legislation, is registered under the number SISGEN A2F33C6. The whole genomes of the lineages have been deposited in the GenBank/NCBI database under the accession number PRJNA1347049.

### Antimicrobial Susceptibility Test

Minimum inhibitory concentrations (MICs) were determined using the broth microdilution method, according to the Clinical and Laboratory Standards Institute (CLSI) guideline M07-A10 [11], employing cation-adjusted Mueller-Hinton broth (BD Difco™, Sparks, MD). The following antimicrobials were tested: meropenem (MER), imipenem (IMI), cefepime (CFP), ceftazidime (CAZ), amikacin (AMI), aztreonam (ATM), ciprofloxacin (CIP), colistin (COL), piperacillin/tazobactam (PIP/TAZ), polymyxin B (PLB). ceftazidime/avibactam (CAZ/AVI), ceftolozane/tazobactam (CTZ/TAZ), and imipenem/relebactam (IMI/REL). Ceftazidime and avibactam were provided by Pfizer Inc. (Peapack, NJ), and tazobactam, imipenem, and relebactam by Merck & Co., Inc. (Kenilworth, NJ). All other antimicrobials were obtained from commercial sources (Sigma Co., St. Louis, MO). The criteria for classifying strains as DTR-PA were resistance to ceftazidime (≥ 32 mg/L), aztreonam (≥ 32 mg/L), piperacillin/tazobactam (≥ 64/4 mg/L), imipenem (≥ 8 mg/L), or ciprofloxacin (≥ 2 mg/L). CLSI. The determination of antimicrobial susceptibility testing was according to CLSI guideline M100-Ed35 [12].

### Detection of Carbapenemase Genes by q-PCR

Carbapenemase-encoding genes were identified using TaqMan-based real-time PCR or SYBR Green-based real-time PCR with melt curve analysis. Detection of *bla*KPC and *bla*NDM was performed using a multiplex TaqMan-based real-time PCR assay, following the protocol established by the Centers for Disease Control and Prevention (CDC) (https://www.cdc.gov/gram-negative-bacteria/media/pdfs/kpc-ndm-protocol-2011-p.pdf? CDC_AAref_Val=https://www.cdc.gov/hai/pdfs/labsettings/KPC-NDM-protocol-2011.pdf). Detection of *bla*SPM and *bla*VIM was performed using TaqMan-based real-time PCR assays, following the methodology described by Swayne et al. [13]. Finally, *bla*IMP was detected using a SYBR Green-based real-time PCR assay with melt curve analysis, employing primers described by Swayne et al. [13], 1× SYBR Green Master Mix (Applied Biosystems), 0.2 µM of each primer (IMP-F CCCACGTATGCATCTGAATTAACAAA, IMP-R CCAAACCACTACGTTATCTTGAGTG), 50 ng template DNA, final volume of 25 µL. Isolates that did not yield amplification of any target gene were classified as carbapenemase non-producers.

### Whole Genome Sequencing and Bioinformatics Analysis

Genomic DNA was extracted from bacterial cultures grown in BHI broth using the Wizard^®^ HMW DNA Extraction Kit (Promega), followed by centrifugation to concentrate and wash. DNA quantification and purity assessment were performed using a Qubit fluorometer (Thermo Fisher Scientific, Mississauga, ON, Canada). Sequencing libraries were prepared using the Illumina DNA Prep kit (Illumina, Inc., San Diego, CA, USA) according to the manufacturer’s instructions, and whole-genome sequencing was performed on the Illumina NovaSeq 6000 platform (OGC, Oxford, UK) using NovaSeq SP reagent kits (300 cycles, paired-end reads).

Approximately 40 million 100-base paired-end reads were generated per isolate. Sequencing quality was assessed using FastQC v0.12.1 [14], and reads were trimmed and filtered using Trimmomatic v0.39 [15] with parameters retaining reads with ≥ 90% of bases sequenced and Phred scores ≥ 33. Genome assemblies were performed using Unicycler v0.4.8 [16], and assembly quality was evaluated with QUAST v5.2.0 [17].

Multilocus sequence typing (MLST) was conducted from assembled draft genomes using the MLST software. Each draft genome (FASTA format) was analyzed against the *P. aeruginosa* MLST scheme retrieved from the PubMLST database [18]. The software automatically identified allelic profiles for the seven housekeeping genes (acsA, aroE, guaA, mutL, nuoD, ppsA, and trpE) and assigned the corresponding sequence type (ST) based on the allelic combination. Genomes with ambiguous allelic calls were manually inspected by aligning the corresponding gene regions to the reference alleles using BLASTn.Antimicrobial resistance genes were identified using the ABRicate v1.0.0 database, and plasmid prediction was performed using MOB-suite v3.1.9 [19]. A phylogenetic tree was constructed based on average nucleotide identity (ANI) using FastANI v1.33 and visualized with iTOL v6. The tree was rooted at the midpoint, and bootstrap values were calculated with 1,000 replicates.

## Results

The 10 *P. aeruginosa* strains selected for genotypic characterization, based on their resistance profiles to CAZ/AVI, CEF/TAZ, and IMI/REL, included seven resistant isolates and three susceptible to at least one of these antibiotics. Regarding the type of clinical specimen, 40% of isolates were obtained from tracheal aspirates, 30% from urine samples, 20% from blood samples, and 10% from bronchoalveolar lavage. All patients (100%) had received prior antibiotic therapy before the isolation of *P. aeruginosa*.

### Genomic Characterization and Sequence Typing of *P. aeruginosa* Isolates

Whole-genome sequencing and genome assembly were performed for all ten *P. aeruginosa* isolates. High-quality paired-end reads were obtained, with Q20 values exceeding 98% for all samples. The total number of bases sequenced per isolate ranged from approximately 6.4 to 7.1 Mb, and the GC content ranged from 65.65% to 66.33% (Supplementary material, Table S1). Genome assemblies demonstrated high contiguity and coverage across the isolates (Supplementary material, Table S1), providing sufficient depth for reliable downstream analyses, including multilocus sequence typing and detection of resistance determinants.

**Fig. 1:**
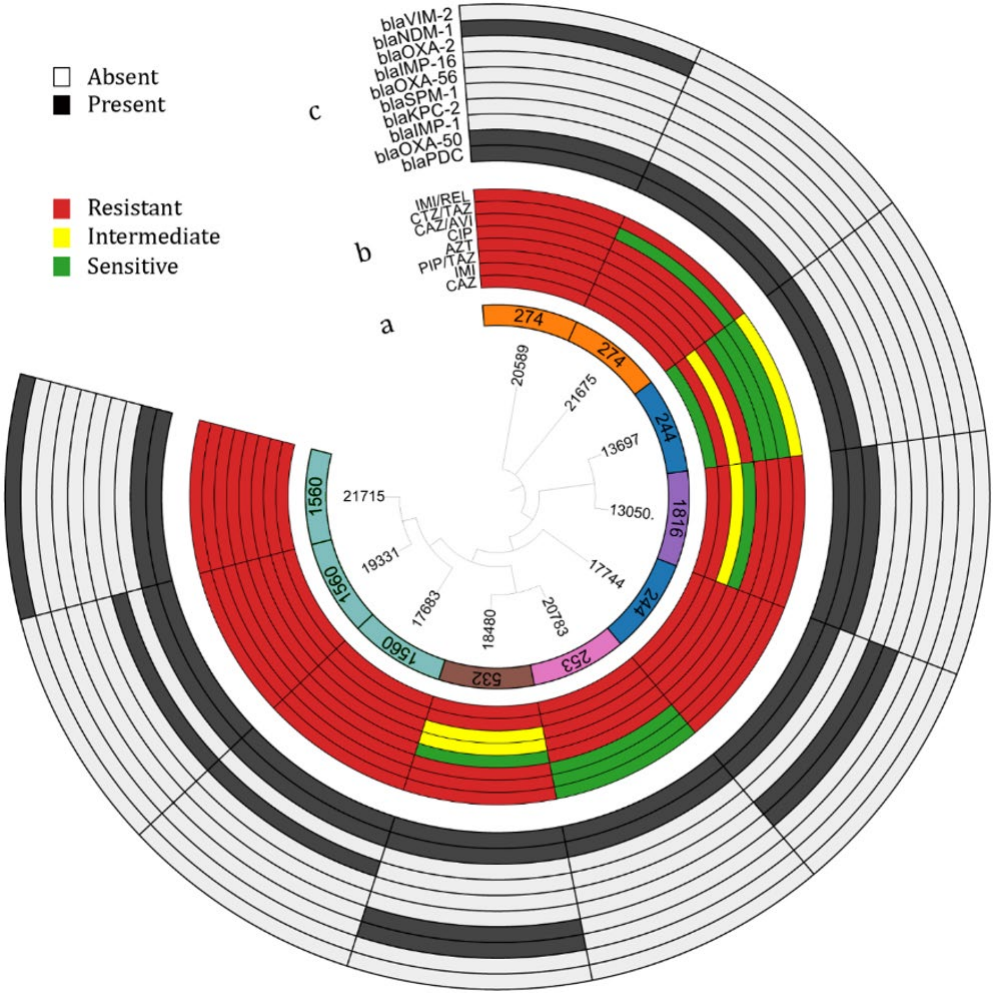
Phylogenetic relationship, sequence types (ST), antimicrobial resistance profiles (CLSI, 2025), and resistance genes of ten *Pseudomonas aeruginosa* clinical isolates from Brazil. In (**a**), the innermost ring represents the ST of each isolate. In (**b**), the middle ring shows the phenotypic resistance profile to selected β-lactams and β-lactam/β-lactamase inhibitor combinations, with colors indicating susceptibility: green = susceptible, yellow = intermediate, red = resistant. In (**c**), the outermost ring indicates the presence of β-lactamase and carbapenemase genes, including chromosomal and plasmid-borne genes. The phylogenetic tree in the center was constructed based on Average Nucleotide Identity (ANI) using FastANI v1.33 and visualized with iTOL v6 (bootstrap values ≥ 70% are shown at nodes; the scale bar indicates nucleotide substitutions per site), illustrating the genetic relatedness among the isolates. This integrated visualization highlights the correlation between sequence type, antimicrobial resistance, and the presence of specific resistance determinants

Multilocus sequence typing revealed that ST1560 was the most prevalent sequence type, accounting for 30% (3/10) of the isolates, followed by ST274, which was represented by two isolates. The remaining sequence types, ST1816, ST244, ST277, ST253, and ST532, were each represented by a single isolate (Fig. 1a).

### Phenotypic and Genotypic Characterization of β-lactam Resistance in *P. aeruginosa*

All isolates were identified as *P. aeruginosa* and exhibited high-level resistance to β-lactams as well as to multiple other antibiotic classes.

Seven of the ten isolates (17744, 20589, 21715, 19331, 17683, 21675, and 20783) were classified as DTR-PA, showing non-susceptibility to all first-line antipseudomonal agents tested (Fig. 1b). In contrast, isolates 13,697, 13,050, and 18,480 did not meet the DTR criteria, exhibiting susceptibility or intermediate responses to at least one first-line antibiotic (Fig. 1a, b; Table 1).

**Table 1:** Antimicrobial susceptibility test. Minimum inhibitory concentrations (MICs) were measured according to the CLSI 2025 guidelines. Red = Resistant; Yellow = Intermediate; Green = Susceptible

ID	SAMPLE SOURCE	DATE OF ISOLATION	RT-PCR	DTR Classification	CFP	CAZ	IMI	MER	PIP/TAZ	AZT	CIP	COL*	POLB*	AMI	CAZ/AVI	CTZ/TAZ	IMI/REL
**17,744**	Blood	05/12/2017	SPM	DTR	> 256	> 128	> 128	> 128	> 256	32	> 16	0,5	0,5	> 256	> 128	> 128	> 128
**20,589**	Urine	17/08/2018	NDM	DTR	> 256	> 128	> 128	> 128	> 256	64	> 16	> 32	16	> 256	32	> 128	> 128
**21,715**	Blood	08/11/2018	VIM	DTR	64	64	> 128	128	128	64	> 16	2	2	256	64	> 128	> 128
**19,331**	Tracheal aspiration	11/05/2018	KPC + SPM	DTR	> 256	64	> 128	> 128	> 256	> 256	> 16	0,5	1	64	> 128	128	> 128
**13,697**	Tracheal aspiration	09/12/2016	IMP	Non-DTR	16	4	16	32	32	64	0,5	1	1	2	4	1	4
**13,050**	Urine	09/09/2016	IMP	Non-DTR	256	128	> 128	128	32	4	> 16	0,5	0,5	> 256	> 128	64	> 128
**17,683**	Urine	04/12/2017	KPC	DTR	> 256	> 128	> 128	> 128	> 256	> 256	> 16	1	1	> 256	16	128	> 128
**18,480**	Tracheal aspiration	01/02/2018	-	Non-DTR	256	> 128	128	64	32	16	≤ 0,06	1	0,5	128	16	> 128	128
**21,675**	Alveolar bronchus lavage	19/10/2018	-	DTR	32	32	32	128	64	128	2	0,5	0,5	32	32	2	8
**20,783**	Tracheal aspiration	21/08/2018	-	DTR	256	> 128	16	32	> 256	128	> 16	> 32	> 32	64	4	2	2

Genotypic analysis confirmed the presence of carbapenemase-encoding genes in seven isolates. Specifically, isolate 17,744 carried *bla*SPM-1; isolate 20,589 carried *bla*NDM-1; isolate 21,715 carried *bla*VIM-2; isolate 13,050 carried *bla*IMP-1; isolate 18,480 carried *bla*IMP-16; and isolates 17,683 and 19,331 carried *bla*KPC-2. Phenotypically, all five MBL-producing isolates were resistant to CAZ/AVI and IMI/REL. Susceptibility to CTZ/TAZ was observed only in the two IMP-producing isolates (13050 and 18480). The two KPC-producing isolates (19331 and 17683) were resistant to all three novel β-lactam/β-lactamase inhibitor combinations (CAZ/AVI, CTZ/TAZ, and IMI/REL). A detailed correlation between the carbapenemase genes and the susceptibility profiles to these novel agents is presented in Fig. 1b and c.

Three isolates (13697, 21675, and 20783) were carbapenemase-negative. All three were susceptible to CTZ/TAZ. Isolate 13,697 was also susceptible to CAZ/AVI and showed intermediate resistance to IMI/REL. Isolate 20,783 was susceptible to both CAZ/AVI and IMI/REL. Isolate 21,675 was resistant to all β-lactams tested, except for CTZ/TAZ. Resistance to polymyxins was observed in two isolates, 20,589 (NDM-1 producer) and 20,783 (carbapenemase-negative), both of which were also resistant to amikacin. Strain 20,783 carried the rmtD gene, which confers high-level resistance to all aminoglycosides (Supplementary Material, Table S1).

The classification of isolates as DTR or non-DTR did not accurately predict susceptibility to the novel β-lactam/β-lactamase inhibitor combinations. Notably, the two IMP-producing isolates were non-DTR because they retained susceptibility to aztreonam (13050) and ciprofloxacin (18480). Furthermore, two carbapenemase-negative DTR isolates (21675 and 20783) were susceptible to CTZ/TAZ.

Plasmid sequence analysis revealed that several isolates harbored carbapenemase genes located on mobile genetic elements. From short-read sequencing data, the following plasmid features were confidently identified: Inc group assignment, plasmid size estimates, and the presence of carbapenemase and associated resistance genes. Specifically, a 7.7 kb IncU-type plasmid carried *bla*IMP-1 in isolate 13,050. In isolates 17,683 and 19,331, *bla*KPC-2 was found on IncU plasmid of 7.7 kb and 15.8 kb, respectively. Isolate 18,480 carried multiple resistance determinants on a large, approximately 252 kb plasmid; however, no canonical Inc group was identified for this plasmid. The plasmid carried *bla*IMP-16, *bla*OXA-2, and the sulfonamide resistance gene *sul1.* The remaining isolates (13697, 17744, 20589, 20783, 21675, and 21715) did not harbor plasmid-borne carbapenemase genes. Due to the limitations of short-read sequencing, complete plasmid circularization could not be confirmed for plasmids larger than ~ 50 kb, and the Inc group of the ~ 252 kb plasmid in isolate 18,480 remains undetermined. Long-read sequencing would be required for complete plasmid reconstruction and full characterization of plasmid architecture. In addition to these acquired carbapenemases, all isolates carried chromosomal *bla*PDC variants (*bla*PDC-1, *bla*PDC-2, *bla*PDC-5, *bla*PDC-7, *bla*PDC-9, *bla*PDC-10, *bla*PDC-195, *bla*PDC-212, and *bla*PDC-216) and intrinsic *bla*OXA-50-like genes (Fig. 1c). Resistance to CAZ/AVI and IMI/REL in the two KPC-producing isolates (19331 and 17683) and in the carbapenemase-negative isolate 21,675 may involve alternative mechanisms, such as porin loss or efflux pump overexpression, although these were not investigated in the present study (Fig. 1b, c).

## Discussion

In this study, we performed genomic and phenotypic analyses of carbapenem-resistant *P. aeruginosa* isolates obtained from hospitals in the states of Paraná and Santa Catarina, Brazil. Our findings provide insight into the molecular epidemiology, antimicrobial resistance profiles, and plasmid-mediated dissemination of carbapenemase genes in Brazilian clinical settings.

The isolates exhibited a heterogeneous population structure, as revealed by multilocus sequence typing (MLST). ST1560 was the most prevalent sequence type, accounting for 30% (3/10) of the isolates, followed by ST274 with two isolates. The remaining isolates belonged to ST1816, ST244, ST277, ST253, and ST532. This diversity highlights multiple circulating clones within the sampled hospitals, reflecting a complex epidemiology of *P. aeruginosa* in these regions. Notably, ST1560 is less frequently reported in global databases compared to high-risk clones like ST235 and ST308, yet it appears to be a dominant lineage within this collection, suggesting local adaptation and potential clonal expansion in Brazilian healthcare facilities [20–22]. The detection of three *P. aeruginosa* isolates belonging to ST1560 is particularly concerning, as they harbor clinically significant carbapenemases: two carry KPC-2 and one carries VIM-2, highlighting the emergence of this uncommon sequence type as a possible reservoir of multidrug resistance determinants.

ST1560 has been identified in Brazil as an emerging endemic lineage associated with XDR phenotypes and the *bla*KPC gene. However, its geographic origin and the plasmid harboring this gene have not yet been characterized, indicating that its dissemination may be driven by local factors related to hospital antimicrobial pressure [23]. Similarly, ST1816, initially reported in Japan and linked to the *bla*VIM-24, *bla*VIM-60, and *bla*VIM-66 variants, has subsequently been detected in Brazil. This pattern likely reflects sporadic clonal introduction followed by limited local circulation, with no current evidence supporting sustained global dissemination [24]. Additionally, ST308 has been observed in Brazil, and its presence may be attributed to regional circulation, as this ST has been reported previously in Colombia and in various regions across South America, North America, Europe, and Asia [25].

In contrast, clones such as ST274, ST235, and ST244 demonstrate extensive international dissemination and are frequently linked to the acquisition of β-lactamase genes via horizontal gene transfer mediated by class 1 integrons [26]. The epidemic ST274 clone, characterized by XDR phenotypes, is distributed across North and South America, Europe, Asia, Africa, and Oceania, and is associated with β-lactamase genes including *bla*IMP, *bla*PDC, and *bla*OXA [27].

The ST235 clone, a pandemic lineage likely originating in Europe in the late 1990s, has emerged in hospital-associated *P. aeruginosa* isolates and possesses a broad array of β-lactamase genes, including *bla*KPC, *bla*SPM, *bla*IMP, *bla*NDM, *bla*VIM, and *bla*OXA. In Brazil, this clone is acknowledged as a significant vector for the dissemination of carbapenemase enzymes [26].

ST532 has been identified in Brazilian isolates from the Southeast and Northeast regions; however, comprehensive data concerning its origin, dissemination, and epidemiological significance remain scarce [27]. ST244 was initially reported in Colombia and is associated with XDR/DTR strains, as well as a plasmid harboring the *bla*KPC gene (*pBH6*). In addition to *bla*KPC, ST244 may carry other resistance genes, including *bla*IMP, *bla*NDM, *bla*VIM, and *bla*OXA. This sequence type has been documented in multiple countries across North and South America, Europe, Asia, and the Middle East, encompassing regions such as the Persian Gulf and India [26]. In Brazil, ST244 represents the second most prevalent *P. aeruginosa* clone in the Southeast and North regions, surpassed only by ST277. It has been predominantly detected in non-clinical settings, including hospital wastewater treatment plants, at various stages of the treatment process, frequently in association with resistance genes such as *bla*VIM and *bla*KPC [27].

Within the Brazilian context, ST277 constitutes the first reported and most extensively disseminated clone to date. Initially identified in an oncological patient in the state of São Paulo, this ST is recognized as an endemic clone that is strongly associated with the dissemination of the *bla*SPM-1 gene across various clinical settings. ST277 has played a pivotal role in the epidemiology of carbapenem resistance in Brazil, particularly among MDR and XDR isolates. Its occurrence outside Brazil remains rare, limited to sporadic reports from Japan, China, and the United Kingdom [27].

Phenotypic analysis revealed a predominance of *P. aeruginosa* with difficult-to-treat resistance (DTR-PA), with seven out of ten isolates classified as DTR according to international definitions [4]. These isolates demonstrated high-level resistance to almost all β-lactams tested, including carbapenems and novel β-lactam/β-lactamase inhibitor combinations, such as ceftazidime-avibactam and ceftolozane-tazobactam. Ceftolozane-tazobactam appeared to serve as a useful indicator of the absence of carbapenemases among carbapenem-resistant *P. aeruginosa* isolates. Nevertheless, the coexistence of other β-lactamases or resistance mechanisms may compromise its clinical efficacy, underscoring the need for confirmation of susceptibility through comprehensive laboratory testing.

Among the non-DTR-PA isolates, isolate 17,744 showed the highest resistance levels, with MICs greater than 128 mg/L for all β-lactams. This isolate belonged to ST277, endemic in Brazil, and harbored multiple β-lactamase genes, including *bla*PDC-212, *bla*OXA-50, *bla*OXA-56, and the metallo-β-lactamase *bla*SPM-1, which together likely explain its extreme resistance. On the other hand, isolate 13,697, belonging to ST244, which is considered high-risk and frequently associated with multidrug resistance, represented the least-resistant phenotype, with MICs within the susceptible or intermediate range, and carried only *bla*PDC-216 and *bla*OXA-50. These observations illustrate the broad spectrum of resistance present even among isolates collected in the same hospital environment.

Interestingly, isolate 20,783 demonstrated resistance to all β-lactams despite lacking any carbapenemase genes other than *bla*OXA-50 and *bla*PDC-195. This suggests the involvement of alternative resistance mechanisms, such as porin loss, efflux pump overexpression, or other chromosomal mutations, which have been described as contributing factors to carbapenem resistance in *P. aeruginosa* [28, 29]. This finding highlights the importance of integrating phenotypic testing with genomic analyses, as the absence of known resistance genes does not necessarily indicate susceptibility.

Genotypic characterization revealed a diversity of carbapenemase-encoding genes. Metallo-β-lactamases, including *bla*NDM-1 (20589), *bla*VIM-2 (21715), and *bla*IMP variants (*bla*IMP-16 in 18480 and *bla*IMP-1 in 13050), were detected, along with class A carbapenemases (*bla*KPC-2 in 17683 and 19331) and *bla*SPM-1 (17744). These genes correlated with resistance to β-lactam/β-lactamase inhibitor combinations, consistent with their known biochemical activities [23]. Notably, the detection of *bla*NDM-1 in isolate 20,589, from a lineage in which NDM-producing *P. aeruginosa* had been exceedingly rare in Brazil before the COVID-19 pandemic, represents a clinically significant finding. Recent surveillance studies have reported a noticeable increase in the occurrence of *bla*NDM among *P. aeruginosa* isolates in Brazil after 2020 [23].

Plasmid analysis revealed that 40% of the isolates harbored carbapenemase genes on mobile genetic elements. Specifically, *bla*IMP-1 was located on a 12.9 kb plasmid in isolate 13,050. The *bla*KPC-2 gene was carried on IncU-type plasmid in isolates 17,683 (7.7 kb) and 19,331 (15.8 kb). The identification of *bla*KPC-2 on IncU plasmids in our collection is notable and corroborates regional findings, such as the report by Tartari et al. (2021) of a small (7.9 kb) IncU plasmid carrying *bla*KPC-2 in an extensively drug-resistant *P. aeruginosa* from Southern Brazil. This highlights the established role of this plasmid family in the dissemination of *bla*KPC-2 among *P. aeruginosa* in this geographical context. Isolate 18,480 carried multiple resistance determinants, including *bla*IMP-16, *bla*OXA-2, and sul1, on a large (252 kb) plasmid for which no canonical Inc group was identified. The presence of carbapenemase genes on plasmids underscores the risk of horizontal gene transfer between strains and across species in hospital environments, facilitating rapid dissemination of resistance. The coexistence of multiple resistance determinants on the same plasmid, as observed in isolate 18,480, underscores the potential for the simultaneous spread of multidrug resistance, which poses significant challenges for infection control and antimicrobial stewardship [30, 31].

The distribution of resistance among the isolates was not uniform. While the DTR strains showed high-level resistance to nearly all β-lactams, intermediate susceptibility to newer agents, such as ceftazidime-avibactam and ceftolozane-tazobactam, was observed in some isolates (20589, 21715, 21675, and 20783), suggesting that these drugs may retain partial efficacy against select strains. However, the plasmid-encoded metallo-β-lactamases likely compromise the activity of these agents in isolates carrying *bla*NDM-1, *bla*VIM-2, or *bla*IMP variants, consistent with previous observations of reduced inhibitor efficacy in MBL-producing *P. aeruginosa* [3].

Our results also illustrate the clinical relevance of monitoring resistance phenotypes even when genotypic data is available. The presence of carbapenem-resistant isolates without known carbapenemase genes (20783) reinforces the concept that alternative resistance mechanisms may drive treatment failures, particularly in environments with high antibiotic pressure. Moreover, isolates such as 13,697 and 21,675, which exhibited limited resistance and did not meet the criteria for DTR, serve as important reminders that not all P. aeruginosa strains circulating in hospitals are highly resistant, and that surveillance should capture this heterogeneity to inform therapeutic strategies more effectively.

Overall, these findings highlight the remarkable adaptability of *P. aeruginosa* in acquiring resistance to antibiotics across multiple classes. The coexistence of multiple β-lactamases within isolates, combined with the diversity of antimicrobial-efflux systems, underscores the complexity of resistance mechanisms and the urgent need for continuous molecular surveillance in clinical settings.

The diversity of genes encoding carbapenemases (SPM, KPC, VIM, NDM, and IMP) detected among these isolates, with 40% (4/10) of isolates harboring at least one plasmid-borne carbapenemase gene, highlights the potential for horizontal transfer of resistance determinants within the hospital environment. The coexistence of multiple resistance genes on the same plasmid, as observed in isolate 18,480, further highlights the risk of disseminating multidrug resistance and underscores the importance of ongoing genomic surveillance to monitor the spread of plasmid-mediated carbapenemases.

From an epidemiological perspective, the co-existence of diverse STs, multiple resistance genes, and plasmid-mediated determinants indicates a complex genomic landscape in Brazilian hospitals. Unlike high-risk international clones, such as ST235, ST233, or ST308, which are often associated with outbreaks in Europe and Asia [32, 33], our data suggest that local lineages, including ST1560 and ST274, may play a prominent role in driving resistance in this setting. This highlights the necessity of region-specific surveillance and molecular epidemiology studies to capture the dynamics of local *P. aeruginosa* populations.

We acknowledge that this study focused primarily on acquired carbapenemase genes; other resistance mechanisms, such as efflux pumps, porin loss, or chromosomal mutations, were not investigated and may also contribute to the observed phenotypes. These mechanisms are currently being addressed in a separate comparative genomics study.

Limitations of the study: We acknowledge several limitations. First, only 10 out of 300 carbapenem-resistant *P. aeruginosa* isolates were selected for genomic analysis. The selection was purposefully based on resistance profiles to novel β-lactam/β-lactamase inhibitor combinations to enable comparative analyses, which introduces a significant selection bias. Therefore, our findings regarding sequence type distribution, prevalence of carbapenemase genes, and clonal diversity are not representative of the broader *P. aeruginosa* population in southern Brazil. Second, the small sample size limits the statistical power and generalizability of our conclusions. Third, due to the use of short-read sequencing alone, complete plasmid assembly and characterization of large plasmids could not be achieved. Fourth, mechanisms such as efflux pump overexpression and porin loss were not experimentally investigated. Future studies with larger, unbiased sampling and long-read sequencing are necessary to comprehensively assess the molecular epidemiology of carbapenem-resistant *P. aeruginosa* in this region.

## Conclusion

Overall, our study underscores several critical points as the predominance of DTR *P. aeruginosa* in the sampled hospitals, with multiple isolates harboring plasmid-borne carbapenemases, the heterogeneity of resistance phenotypes, including the presence of resistant strains lacking known carbapenemase genes, the risk of horizontal dissemination of resistance determinants via plasmids and the importance of integrated genomic and phenotypic surveillance to guide infection control and optimize therapeutic strategies. These findings have important implications for clinical management, antimicrobial stewardship, and public health policy in Brazil, highlighting the ongoing challenges posed by multidrug-resistant *P. aeruginosa* in hospital environments.

## Electronic Supplementary Material

Below is the link to the electronic supplementary material.

Supplementary Material 1

## Data Availability

All data supporting the findings of this study are included in this published article and its supplementary information files.
