# Supplementary material for: Genomic and Phenotypic Insights into Carbapenemase-Mediated Resistance and Clonal Diversity of Pseudomonas aeruginosa Clinical Isolates from Southern Brazil
Source: Curr Microbiol. 2026 Jun 18;83(8):438. doi: 10.1007/s00284-026-05023-9 (PMC13279751; doi:10.1007/s00284-026-05023-9)
Supplement: Supplementary file 1 — Supplementary Material 1 [file 284_2026_5023_MOESM1_ESM.docx]

**Table S1.** Genomic statistics of sequenced clinical isolates of *Pseudomonas aeruginosa*.

| **Sample** | **Contigs*** | **Genome size (bp)** | **N50 (bp)** | **GC %** | **Coverage X** |
| --- | --- | --- | --- | --- | --- |
| 13050 | 32 | 6,809,043 | 645,601 | 66,04 | 424,2 |
| 13697 | 36 | 6,612,233 | 459,172 | 66,12 | 440,2 |
| 17683 | 123 | 7,146,924 | 179,791 | 65,65 | 683,4 |
| 17744 | 37 | 6,853,273 | 499,836 | 66,03 | 533,7 |
| 18480 | 79 | 6,906,970 | 230,128 | 65,7 | 487,9 |
| 19331 | 75 | 6,931,955 | 280,682 | 65,87 | 453,1 |
| 20589 | 51 | 6,909,516 | 428,517 | 66,09 | 362,2 |
| 20783 | 113 | 6,882,445 | 135,498 | 65,92 | 404,6 |
| 21675 | 61 | 6,434,588 | 258,550 | 66,33 | 615,0 |
| 21715 | 75 | 6,880,350 | 335,128 | 66,06 | 570,1 |

* Considering only contigs with size greater than or equal to 1,000 bp.
